# Needs of parents during their intrauterine or perinatal child loss – a qualitative multi-method study

**DOI:** 10.1186/s12889-026-27687-5

**Published:** 2026-05-12

**Authors:** Julian Siepmann, Lydia Bauernfeind, Daniela Nuber-Fischer, Christian Rester, Karsten Gensheimer, Fritz Sterr

**Affiliations:** 1 Deggendorf Institute of Technology, Faculty of Applied Healthcare Sciences, Land-Au 27, Deggendorf, 94469 Germany; 2 Munich, Germany; 3 Faculty of Health, School of Nursing Sciences, Witten/Herdecke University, Witten, Germany

**Keywords:** Child loss, Fetal death, Miscarriage, Parents, Pregnancy loss, Qualitative study, Stillbirth

## Abstract

**Background:**

The loss of a child during pregnancy or birth is a profound and enduring experience that deeply shapes the lives of families. However, little is known about how parents experience the immediate moment of loss, including the diagnosis and medical procedures in the hospital. As a result, there is a lack of guidelines and recommendations for professionals on how to appropriately support affected parents.

**Aim:**

To explore the needs of parents during their intrauterine or perinatal child loss.

**Methods:**

We conducted a qualitative multi-method study with world cafés and focus groups in 2025. In this study, 16 German-speaking parents who had lost their child during pregnancy in the last 12 months took part. Data was collected using field notes, photos and audio records. Analysis of data followed an inductive thematic analysis by Braun and Clarke, which was performed by two researchers.

**Findings:**

The analysis revealed the three principal themes ‘experiencing implications of child loss’, ‘emerging needs and demands related to child loss’ and ‘individual strategies to cope with the situation’. The needs were diverse and included the requirement for social support, demands on healthcare professionals and adjustments to administrative processes. In addition, the importance of creating memories with the child was emphasized, as was the need for safety and protection, having time and pausing, and being in control. Finally, the need to be strong, to be involved and to participate was also emphasized, as was understanding one's own situation and overcoming tensions.

**Conclusion:**

Parents during intrauterine or perinatal child loss show complex needs. These must be addressed to prevent negative consequences. Healthcare professionals are responsible for the course of parents’ treatment and linked outcomes.

**Clinical trial registration:**

ClinicalTrials.gov Identifier NCT06771661, registered on January 13th, 2025.

**Supplementary Information:**

The online version contains supplementary material available at 10.1186/s12889-026-27687-5.

## Background

The loss of a child during pregnancy or at birth represents a devastating and often life-altering experience for parents. Despite a global decline in stillbirths over the past 30 years, the overall number of pregnancy and birth-related child losses remains high [1]. Although definitions of stillbirths and methods of data collection vary across countries and healthcare systems, the global number of stillbirths is estimated at around two million per year [2]. On a global scale, there is a pronounced difference in stillbirth rates, with developing countries reporting 25.5 per 1,000 deliveries compared to 5.3 per 1,000 in developed countries [3]. Although at a lower level, even in a developed country like Germany the stillbirth rates show an upward trend again between 2010 and 2021 [4].

Up to this point, a broad spectrum of risk factors has been identified that can contribute to a pathological course of pregnancy. In addition to an advanced maternal age and ethnic background, studies found that malnutrition, fetal growth restriction, infections, cervical pathology, as well as maternal mental disorders and substance abuse represent key determinants associated with an increased risk of fetal loss during pregnancy [5–7], while in more than 60% of cases, the etiology of fetal demise remains unexplained hut [8].

Beyond its clinical implications, intrauterine or perinatal loss carries profound psychosocial consequences for involved parents [9] and siblings [10]. The importance of adequate support and sustained psychological processing following traumatic pregnancy loss is underscored by existing studies, offering insights into the profound psychological and physical consequences associated with this experience [11, 12]. Affected parents grieve in different ways and have different tasks during this process [13]. Unravelling their grief of child loss is hard work for those affected [14] and, if left unaddressed, can become chronic grief [15]. The loss can also manifest itself in post-traumatic stress symptoms, anxiety, and depression, which not only restrict those affected in their everyday lives, but also have an impact on subsequent pregnancies [16]. Trauma-informed care is considered a key intervention in supporting parents during this period, and specific recommendations are made, such as screening for trauma, low-threshold access to care, individualized services, consistent and continuous support, and specialized training for healthcare professionals (HCPs) [17].

However, while existing studies address the long-term consequences of pregnancy loss, a gap remains in research on how affected parents experience the immediate acute moment of loss – for example, within the hospital setting. Moreover, a review of the medical and nursing literature reveals that neither clinical guidelines nor textbooks provide recommendations on how to adequately support affected parents in the acute moment of loss. This is also evident in the lack of systematic assessment of potential trauma in pregnant women to date [18]. Yet such guidance in the immediate aftermath of perinatal or intrauterine child loss appears highly relevant in light of the potential consequences and the need for their prevention.

## Aim and research question

Based on this, the aim of our study is to explore and depict the acute needs parents have when losing their child in pregnancy or during birth. Thereby, we followed the research question: “What needs and requirements arise for families experiencing intrauterine or perinatal loss of their child?”

## Methods

This sub-study is embedded in a larger mixed-methods study [19]. To answer the underlying research question, we conducted a qualitative multi-method study. This was particularly suitable as the combination of different methods enabled a versatile and in-depth examination of parents’ needs. The reporting of this study follows the ‘Standards for Reporting Qualitative Research’ guideline (SRQR) [20].

### Qualitative approach and research paradigm

In this study, we conducted a phenomenological approach to gain an in-depth understanding of parents’ needs that was reported firsthand by those affected [21, 22]. In doing so, we have followed a constructivist logic. It is assumed that parents do not simply passively absorb information and knowledge but actively construct it themselves. In this way, they develop their own, self-constructed understanding of their situation and environment [23, 24].

### Researcher characteristics and reflexivity

All researchers that were involved in this study are registered nurses and have worked with parents after intrauterine or perinatal child loss, either in intensive care units (CR, FS), pediatric intensive care units (LB) or psychiatry wards (JS, KG). Since the entire study is designed as participatory research, an affected mother (DNF) has also been involved in the research process from the outset, accompanying the team with her own reflections and the experience from several years of active counseling of affected parents.

The data analysis was primarily carried out by JS and FS. With his focus on psychiatry, JS has a broader and more nuanced perspective on the psychological experiences of parents. Due to his professional background, FS has a stronger focus on physical experiences and needs. To reconcile and expand these perspectives, DNF was repeatedly involved in the analysis and key findings were discussed with her.

### Sampling strategy

Affected parents (mothers, fathers) were included in the study if they were at least 18 years old and could speak and understand German. To account for the influence of temporal distance on processes of narrative reconstruction and meaning-making, we included only participants whose experiences occurred within the preceding 12 months [25–27]. They also had to participate voluntarily and be in a stable psychological condition.

The passive recruiting of participants followed a snowball sampling and took place primarily via social media. The study was publicized in posts and stories by the university conducting the study and the participatory researcher. In addition, various influencers who provide information on the topic of child loss during pregnancy or childbirth were informed about the study. Most of these also shared the information. Finally, regional healthcare facilities were informed about the study and sent flyers.

If participants were interested, they had to actively contact the researchers themselves. A virtual meeting was held with each interested party in advance, in which the objectives of the study, the inclusion criteria and the opportunities for participation were discussed. In this context, the psychological stability of the participants was also explored. It was assessed based on the participants’ self-reports and further supported by the transparent information provided about the study.

### Characteristics of participants

After recruiting 22 parents for our study, six people canceled on short notice for the first collection day due to illness (winter, flu season). In the end, 16 affected parents, including ten mothers and six fathers, took part in this study. Their child loss was on average 5.69 months ago. Fourteen of the participants lost their child during pregnancy and 87.5% of them gave birth in hospital. The women were between 8 and 37 weeks pregnant at the time of their loss. While 13 individuals lost their child unintentionally, three parents terminated the pregnancy deliberately for various reasons. Further characteristics of the study participants can be found in Table 1.

**Table 1:** Characteristics of included participants

	Study participants (*N* = 16)
Sex	
Female, *n* (%)	10 (62,5)
Male, *n* (%)	6 (37,5)
Age, years (min-max)	35.06 (30–42)
Female, years (min-max)	34.5 (30–42)
Male, years (min-max)	36 (31–38)
Time since loss, months (min-max)	5.69 (2–12)
Stage of loss	
During pregnancy, n (%)	14 (87,5)
During birth, n (%)	2 (12,5)
Week of loss, weeks (min-max)	24.19 (8–39)
1. Trimester, n (%)	2 (12,5)
2. Trimester, n (%)	8 (50,0)
3. Trimester, n (%)	6 (37,5)
Location of loss	
Hospital, n (%)	14 (87,5)
Home, n (%)	2 (12,5)
Termination of pregnancy	
Unintentionally, n (%)	13 (81,25)
Intentionally, n (%)	3 (18,75)
Further children	
No, n (%)	10 (62,5)
Yes, n (%)	6 (37,5)

### Ethical reflections

As parents after child loss are a vulnerable group [28, 29], an ethical reflection of the research project was fundamentally important. Our primary goal was to prevent any potential re-traumatization, given the sensitive nature of our study. To this end, we have drawn up a comprehensive application for an ethical assessment, including an emergency plan for stressful situations and following the Declaration of Helsinki [30]. Throughout the entire study period, a psychiatrist working at the university as well as a professor of psychiatric nursing with practical clinical expertise were available as contact persons in cases of high emotional distress. In November 2024, we received a positive ethics vote (Number: GEHBa-202409-V-237-R2) from the Joint Ethics Committee of Bavarian Universities (GEHBa). In addition, we have developed a plan for the special protection of participant data and concluded this with the data protection officer of the university conducting the study. According to this, each participant must sign an informed consent and confirm that his/her participation is voluntary in order to take part in the study. Detailed information can be found in the study protocol [19].

### Data collection

To do justice to the complexity of the experience, we have opted for a multi-method approach embedded in a co-creative design [31]. Specifically, we invited affected mothers and fathers to one of two data collection days at a central location in Munich, Germany that was both comfortable and reserved for the participants. On each of these days, we worked for six hours in a world café [32] and a focus group [33] on the underlying research question. The interview guide for the focus groups was newly developed for this study and is to be found in Supplement 1.

The World Café was divided into three 30-minute sessions. At the first station, participants were asked to place a total of 31 needs that we had identified in studies (e.g., sensitivity of HCPs, professional competence, continuous communication) on a scale. This scale was arranged twice; first, participants sorted the needs along their relevance. Second, they had to rank them according to how the needs were actually considered in their moment of loss. What was interesting for the researchers here was not only the result, but above all the discussion about the final position and finding a consensus.

At the second station, 24 excerpts from newspaper articles and reports on pregnancy loss were placed in the middle of a circle of chairs. The participants were asked to take one to three articles and comment on what they found good or bad about them and how they thought the loss should be reported.

At the third station, 48 picture cards depicting different symbols (e.g., lion, scales, Justicia, cube, sand, sun, mountain, handshake) were placed in the middle of participants. These were asked to choose one or two cards and use them to formulate an impulse about what an ideal type of care would look like for them during the loss.

We documented the world café with field notes and took photos of the results at the first station. In addition, we recorded the audio of the group discussion and created field notes. Detailed information on these methods is reported in the study protocol [19].

### Data analysis

Once the data collection was complete, we transcribed all audio files and field notes verbatim and exported them as separate, pseudonymized files. Together with the photos, these formed the basis for the data analysis and triangulation that we performed in MAXQDA. For this purpose, we used the thematic analysis according to Braun and Clarke [34–37]. This method is particularly suitable because it allows media items such as photos to be included in the analysis. Moreover, it goes “beyond the semantic content of the data and […] to identify or examine the underlying ideas, assumptions and conceptualizations” (34, p. 84).

Braun and Clarke are seeking for central themes in their analyses, which they define as “something important about the data [that] represents some level of patterned response or meaning within the data set” (34, p. 82).

After we defined the data set, we followed the recommended six steps to conduct an inductive thematic analysis [34]. Initially, we familiarized ourselves with the data and generated initial codes at different levels. Next, we searched for themes by ourselves and in the research group. Following this, we reviewed the identified themes, defined and named them. Finally, we report these themes, their sub-themes and original quotes in the findings of this article. In accordance with the method, we also considered the checklist for a good thematic analysis [34]. A total of 15 criteria is assigned to the transcription, coding, analysis, overall and written report.

### Enhancing trustworthiness

Several methods were used to confirm our thematic analysis. On the one hand, we triangulated all data and checked for consistency and contradictions. In doing so, two researchers (JS, FS) independently analyzed the transcripts and photos. These were subsequently synthesized in a discursive conversation. In the event of inconsistencies, a third researcher was involved (LB). On the other hand, the analyses were presented to the participatory researcher (DNF). Together, these were checked for comprehensibility. In addition, two social scientists at our faculty who were not involved in the data collection and analysis also checked the results and fed questions back to the researchers.

## Findings

### Analysis of parents’ needs

Our thematic analysis identified three principal themes as to the phenomenon of living through the early stages of child loss. The first principal theme of *Experiencing implications of child loss* offers insights into the burdensome situation faced by bereaved parents, outlining a wide range of psychological, social, and existential implications that serve as preconditions for the needs that subsequently emerge. The second principal theme *Emerging needs and demands related to child loss* further expands on the notion of dealing with the loss of a child, by identifying and describing the evolving needs and requirements that arise in parents throughout the process. The third principal theme *Individual strategies to cope with the situation* adds another perspective by highlighting the personal strategies and measures parents employ to manage and endure the loss of their child. Table 2 provides an overview of principle themes and sub-themes.

**Table 2:** Overview of principal themes and sub-themes

Principal theme	Sub-themes
Experiencing implications of child loss	Overwhelming mental load
	Having physical symptoms during loss
	Being confronted with a sudden change
	Being confronted with own assumptions
	Specific roles and adaptions
Emerging needs and demands related to child loss	Requiring social support by others
	Need for safety and protection
	Being in control
	Will and need to be strong
	Demands on HCPs and healthcare
	Need for involvement and participation
	Adjustment of administrative processes
	Overcoming tensions in care
	Longing for an understanding
	Importance of creating memories with the child
	Importance of pausing and having time
Individual strategies to cope with the situation	-

#### Theme 1: Experiencing implications of child loss

##### Overwhelming mental load

Parents´ needs and requirements are often embedded into several determining factors on a psychological and social level. First and foremost, parents experience an overwhelming mental load by the occurring demands and go through a range of alternating emotions. The high levels of emotional vulnerability are determined by feelings of guilt and self-condemnation and further aggravated by the notion of being pressured into decisions as well as a constant feeling of uncertainty.

„*Because often*,* especially in the beginning*,* there’s such an overload of information — you’re told everything at once*,* like who will handle the burial*,* where the baby will be taken*,* maybe there’s even a bereavement photographer — and it’s just all a bit too much.”* (M001: 630–633).

In this instance, a mother recounts the overwhelming demands she faced within the clinical setting in the immediate aftermath of her child’s death. Having to consider all these aspects when already being burdened by the loss leaves parents emotionally exposed and vulnerable. It becomes apparent that the considerable mental load is not due to a singular stimulus but rather a wide range of influences on an emotional level, forcing those affected to make decisions at various levels.

##### Having physical symptoms during loss

Besides the overwhelming mental load, parents report that child loss also implies physical symptoms such as pain, sleeplessness, and lack of appetite. Parents appear to have an increased body awareness and detect changes and abnormalities as well as interpret them and find their own reasoning.

##### Being confronted with a sudden change

Parents’ overwhelming mental and physical strain is largely attributable to the sheer suddenness of events, as they are often caught off guard by the distressing news they receive. When confronted with a sudden change, parents feel exposed to a situation they have never faced before and for which they feel ill-prepared to cope.

##### Being confronted with own assumptions

Another determining factor in parents’ needs and requirements is their perception of the motives and competencies of the professionals they interact with. In the context of child loss during pregnancy or birth, these perceptions and interactions are often shaped by parents’ own assumptions, which tend to be predominantly negative.

*“That’s their daily business.”* (M011) – *“Yes*,* like –like how often they issue that scrap of paper.”* (M018) – *“It’s just routine for them*,* but for you*,* it’s not-“* (M011) – *“For you*,* it’s your life.”* (M018) (M011/M018: 209–218).

This interaction from one of the focus groups illustrates parents’ implicit expectations towards professionals’ attitudes and empathy. Disappointment arises when HCPs adhere to their routines in a prosaic manner while parents are experiencing an existential crisis. In addition, parents also report experiences in which their initial assumptions were not confirmed, as in these instances negative expectations were countered by a more empathetic approach from healthcare providers.

##### Specific roles and adaptions

Another crucial implication of child loss is the struggle parents face in adapting to their existing roles while also coming to terms with new role expectations. In this context, interviewees describe situations in which they strive to be recognized as parents but feel denied this form of acknowledgment by others. Furthermore, they struggle to define their role in social interactions, often feeling self-conscious and hesitant to open up when approaching others. Within these specific roles and adaptations, parents also highlight the particularities of experiencing child loss from a male perspective.

*„Yeah*,* I wasn’t really involved. I was there*,* but I don’t know — if you had needed anything and said so*,* I would have been there. But otherwise*,* I wasn’t really involved*,* no.“* (V007: 468–470).

The men in our study often reported feeling less involved in the process than their partners, experiencing a sense of helplessness and perceiving their own needs as less important in these situations. In summary, the first principal theme offers insight into the profound and rapidly changing realities faced by parents after losing a child during pregnancy or at birth. The described phenomena can be understood as underlying conditions that give rise to the needs and requirements of parents facing such burdensome situations.

#### Theme 2: Emerging needs and demands related to child loss

##### Requiring social support by others

Consistent with our research question, the second principal theme explores the specific needs parents encounter while dealing with perinatal loss. In this context, parents identify social support as one of their primary needs. Parents´ lived experiences of receiving support range from having one´s need for social support largely met by family and friends to the notion of feeling alone and having no one to talk to.

A strong desire for guidance and meaningful exchange with others becomes apparent among parents. The interviewees also reflected on the appropriateness of social support, noting that some advice from family and friends was perceived as unhelpful, while a sensitive approach and thoughtful wording were appreciated. Furthermore, a trusting and caring partnership appears to be crucial:

*“For me*,* it definitely helped a lot to go through it together. That’s why I can hardly imagine having to carry all the grief and pain alone—and on top of that*,* facing a partner’s defensive attitude.”* (M016) – *“That’s exactly how it was. Or still is.”* (V008) (M016/V008: 452–456).

In this exchange, the interviewees elaborate on the situation of a father whose partner had ended the relationship with him during the pregnancy, facing the additional burden of lacking a caring partnership as a source of support.

##### Need for safety and protection

Within the narrative of social support, parents express the need to feel protected and guided during this challenging time. They describe themselves as emotionally vulnerable, exposed to an unfamiliar environment and to strangers, feeling insecure and lacking control over the situation. In addition to a sense of protection, they value reassurance and encouragement in their decisions and approach. In many cases, receiving a second opinion on the next steps provides further reassurance and strengthens their confidence.

##### Being in control

In this theme, parents elaborate on the concepts of control and autonomy being subject to change. As to these overarching phenomena, parents desire to retain control, to act autonomously and to make individual and self-assured decisions or at least having the sense that they can do so. Feeling positioned in a place of power and strength with the ability to decide what is best themselves, their body and their child is of great importance for those affected. Yet, this notion is contrasted by a reality where they often find themselves situated within a system of dependency that limits individuals’ ability to act with full autonomy, this adding to the ambivalence of striving for control but not being able to achieve it. In the clinical context, they sense a high degree of external control where they feel the need to enforce their rights against the external structure. In many cases, parents are also not fully aware of their rights, underlining the need for advocacy by others as well as more detailed information.

##### Will and need to be strong

In spite of the burdensome situation, parents still try to maintain strength, attempting to appear strong and clear-minded in front of others. On one hand, parents describe the narrative of staying strong as a necessity, for example in moments they have to enforce their perceived rights or care for others around them (other children, partner or relatives). On the other hand, keeping their strength serves them as a means to restore personal integrity, with parents not only having to stay strong, but also wanting to do so for their own self-perception.

##### Demands on HCPs and healthcare

Another crucial finding is the demands parents place on HCPs. In this context, parents distinguish between the responsibilities of HCPs, knowing who to turn to in specific situations. Being aware of the limited possibilities of HCPs, they refrain from unrealistic demands but still voice distinct expectations. Additionally, there is a strong desire for sensitive interactions, with emotional competence and appropriate language highly valued by parents:

*“So basically*,* a more detailed structure for the staff — I think that would be the most important thing. And that should also include the little things that might seem minor to the clinic staff but are actually extremely significant to us. For example*,* saying something like »Well*,* you’re still young*,* you can always try again« — things like that shouldn’t be said. So*,* really going into detail here. I think that would be something incredibly important.”* (M007: 655–658).

This interviewee highlights the importance of staff using sensitive language and emphasizes the need for HCPs to be specifically trained in interacting with bereaved parents.

Moreover, HCPs are seen as advocates, representing the interests of parents within the healthcare system. In this advocacy role, they relieve parents of many responsibilities - a role closely tied to high levels of trust and strong expectations. Parents affected by child loss also express a need to be acknowledged, to be given time, and to engage in conversations as equals. An open and honest assessment of the situation by HCPs is particularly valued. Overall, the relationship between HCPs and parents emerges as a pivotal element in the continued care following the loss.

##### Need for involvement and participation

In the context of HCPs’ advocacy, parents reflect on their need for involvement and participation in decision-making processes - a need they often perceive as unmet in hindsight: When confronted with crucial decisions, parents often feel excluded from planning the path ahead. From this perspective, the concept of shared decision-making seems even more important for enabling parents to feel actively involved at every step.

##### Adjustment of administrative processes

Closely connected to the demands placed on HCPs is parents’ desire for improvements in administrative processes. Many experience bureaucratic obligations as an additional burden, as navigating the complexities of the healthcare system adds further pressure during an already vulnerable time. This highlights parents’ clear need for administrative, organizational, and bureaucratic procedures to be adapted in a way that is both individualized and sensitive to their situation.

##### Overcoming tensions in care

Another major finding concerning parents’ needs is the perceived ambivalence between normalization and individualization in the context of child loss:

On the one hand, parents wish to be treated as normal, wanting their situation to be seen as acceptable and free from stigma:

*“Normality helped me so much. The way it was handled — that this was just a completely normal birth*,* that I was going to welcome my little one*,* and everyone acted as if I was simply giving birth to a child in the 39th week. For everyone*,* it was so clear and normal*,* nothing terrible was happening*,* and there was no guilt. It was just a completely normal story from start to finish*,* and that helped me tremendously.”* (M018: 46–50).

Based on this assessment, a key expectation parents have of HCPs is to help establish and maintain this sense of normality.

On the other hand, this sense of normality is often disrupted or denied, as child loss remains largely hidden, socially unacknowledged, and underrepresented in collective and institutional narratives. From the parents’ perspective, public perception often fails to reflect the reality of their experience.

This desire for normality is complemented by the need for individualized care: parents frequently express the wish to be separated from other parents — such as mothers who have given birth to healthy children — or to receive distinctly different treatment:

*“Would it maybe be helpful to create a special ward — or a designated space*,* so to speak — because some people mentioned that they heard the crying babies around them while they themselves didn’t have any? Having a place where you can be somewhat on your own*,* without hearing the other babies crying*,* might have helped some of them.”* (V008: 573–576).

The suggestion to be separated from the maternity ward underscores parents’ preference for individualized care, which must be balanced with their desire for normality. Yet, this ambivalence can also be understood as a call for personalized, empathetic, and responsive care that avoids rigid pathways and generic protocols, and instead offers adequate, sensitive, and meaningful support. Parents’ preferences regarding care after perinatal child loss fluctuate along a continuum between normalization and individualization, requiring HCPs to remain attentive to this dynamic and adjust their approach accordingly.

##### Longing for an understanding

Another theme expressed by parents is their longing for a deeper understanding of their situation: Confronted with a sudden change in unfamiliar circumstances, parents strive to make sense of the events they have experienced. From the moment they are informed about the loss of their child, a number of urgent questions emerge that must be integrated into their personal understanding and sense of self. In their effort to make sense of the burdensome situation, parents express a strong desire to be informed about all aspects of what is happening, actively seeking detailed knowledge about both their child and their own situation:

*“Absolutely. Preventive education would have been really helpful. We just stood there thinking*,* »What now? This actually happened to us«*,* and there really wasn’t any possibility or preparation for this to happen.”* (V007: 970–971).

In this instance, a father reflects on how being informed about the possibility of perinatal child loss early in the pregnancy would have been beneficial. When receiving too little information though, parents often feel overwhelmed and uncertain about what is right and important for them in that moment. In the absence of sufficient guidance from staff, they take an active role in seeking out information themselves — basing their understanding on their own assumptions, personal research, or conversations with others, such as friends or relatives.

The desire to make sense of the experience can be divided into two aspects: a cognitive, rational understanding on the one hand, and an emotional understanding rooted in spiritual questions about the deeper meaning of what happened on the other. This longing for an understanding highlights the responsibility of HCPs to guide those affected, both by providing comprehensive information and by offering emotional support.

##### Importance of creating memories with the child

Another essential need expressed by parents is the opportunity to create memories with their child. To get to know their baby and connect with it through all their senses, they try to make the most of every moment. Feeling like a family with their deceased child, even if only briefly, is described as deeply meaningful. Many parents find comfort in symbolic rituals as part of the farewell process. However, the urge to create memories often requires individual initiative and must be frequently voiced — either by the parents themselves or by others acting as advocates. This highlights a critical responsibility for HCPs: to proactively facilitate memory-making in the limited time available, recognizing that parents are generally unprepared for the situation.

##### Importance of pausing and having time

In contrast to the suddenness, haste, and urgency of preceding events, parents also articulated an additional need: the opportunity to pause and cognitively and emotionally process the situation. The experience of being overwhelmed and compelled to make rapid decisions frequently gives rise to the need for psychological processing, including adequate time for reflection and emotional regulation:

*“And that maybe these things could be addressed one after the other — since you usually stay in the hospital for a day or two unless you leave earlier — so that you have some time to let things sink in and clarify a few things.”* (M001: 633–635).

As this statement shows, the necessity to recover and heal does not refer to a long-term perspective but rather to the immediate aftermath of child loss. The desire to pause and reflect may also be interpreted as a responsibility for HCPs to facilitate a certain degree of distance, allowing parents to momentarily detach from the immediacy of events.

In summary, this second principal theme proves to be closely linked to our main research question as it illustrates the diverse and multifaceted needs and demands of parents affected by perinatal child loss. While some parental needs may initially appear contradictory, they often reflect the complexity and ambivalence of the grieving process following the loss. From the perspective of HCPs, the findings point to several clear tasks and responsibilities for supporting parents.

#### Theme 3: Individual strategies to cope with the situation

Apart from having specific demands and expectations concerning the nature of care provided by HCPs, parents also reflect on their own strategies to cope with the loss. One way of dealing with the burdensome events is to find a certain level of detachment by engaging in a distracting activity.

*“And yet*,* for example*,* I sing in a choir*,* and we had a concert. I still went to the concert and sang*,* even though I had concerns about – oh*,* someone might see me there. In fact*,* that weekend - when the birth was on Monday morning - I even went to a wedding.”* (M003: 727–730).

In this instance, a bereaved mother highlights the significance of engaging in joyful activities as a means of coping with her grief. Another strategy employed by parents to achieve a sense of detachment involves the deliberate reduction of external stimuli, for example, by abstaining from social media content connected to pregnancy and child loss.

*“Yes*,* definitely. I actually (laughs) deleted Instagram from my phone a few weeks before because I was overwhelmed by all the pregnancy content being pushed to me. I even told my husband I was tired of getting all that constant pregnancy stuff.”* (M006: 715–718).

The urge to distance oneself from depictions of pregnancy underscores the influence of an ever-expanding media landscape, often perceived as lacking quality control or sensitivity to the needs of bereaved parents, and therefore as offering little support.

Parents also explore a range of additional strategies to address their specific needs, either finding ways themselves or by solutions provided to them by others. This context reveals another important finding: when interacting with HCPs, parents often have to take matters into their own hands to implement their coping strategies.

*“On one hand*,* we actually put up a sign—we had talked about the knight guarding the door earlier (laughs)—so at least the door was ‘protected.’ We created our own space where we could retreat from the usual hospital routine.”* (V006: 496–499).

In this instance, an interviewee elaborates on the measures he and his wife had to take to withdraw from the busy environment of the hospital ward. This finding aligns with the broader sentiment that parents are expected to remain strong in times of grief, highlighting the need for HCPs to take a more active role in advocating for the needs of affected parents.

In summary, this third principal theme adds to a deeper understanding of the phenomenon by illustrating the self-initiated strategies parents employ to manage the mental strain associated with the overwhelming events they have experienced.

## Discussion

This qualitative multi-method study aimed to explore the needs of parents affected by child loss during pregnancy or birth. In this regard, we recruited participants in German-speaking countries and conducted world cafés and focus groups. The subsequent thematic analysis of collected data revealed a total of three principal themes.

Affected parents are *experiencing implications of child loss*. They describe *emerging needs and demands related to child loss*, and parents develop or receive *individual strategies to cope with the situation.* These themes are closely interlinked. While the experiences form the foundation, differentiated needs develop from them. Parents can then either deal with the needs themselves or obtain coping strategies from others.

However, the breadth and scope of the themes differ markedly. Needs were described comprehensively as the largest theme and are differentiated in the analysis. Experiences and coping strategies are differentiated in parts, but only superficially in others and therefore limited in their analytical and informative value. This is due to the fact that the focus of this study was placed on parents’ needs. The latter two themes will be explored in the next step of this mixed-methods study using narrative interviews [19].

Within the principal themes, parents’ experiences and narratives appear heterogeneous. Descriptions of the same theme can be positive, neutral and negative. This becomes clear in the example of the sub-theme ‘Being in control’. While some report being completely controlled by others, others state that they were having the ability to decide (always or in parts). These heterogeneous findings underline the relevance of individual themes. In their narratives, the parents not only reflect the actual needs, but also their characteristics and what they should ideally be like. This also means that the desired response to needs must be negotiated with parents and HCPs on an individual level.

The heterogeneous needs and experiences may also be related to the heterogeneous sample. This study included female and male participants between the ages of 30 and 42 who lost their child at different stages of pregnancy or during childbirth, at home or in the hospital. In addition, we included participants who experienced intentional and unintentional termination of their pregnancies. The deliberate combination of these groups in the study not only broadens the perspective on the phenomenon studied but also supports the destigmatization of a group that continues to be stigmatized.

Data analysis also reveals the various areas of tension that need to be reconciled. Parents long for the normalization of stillbirth and at the same time demand individual care. In addition, parents require adequate guidance in an unexpected, vulnerable situation and simultaneously want freedom of choice and autonomy. These tensions need to be overcome in a joint negotiation process with HCPs and parents.

The data also shows that various experiences and the development of certain needs are intertwined. Parents are confronted with a sudden change and are emotionally overwhelmed by the many simultaneous demands. This leads to their need for safety and protection, but also advocacy and guidance. Another example is the suddenness of bad news and the limited time available either during or after birth. These cause parents to develop a great need for pausing and reflecting. They long to create as many memories as possible with their child in the restricted time available. Awareness of these causal chains enables HCPs to meet parents at an early stage and provide preventative support.

For affected parents, the loss of their child also has considerable financial consequences, which are not described by the parents in the available data, but are recorded in other research [38]. This may be related to the fact that several issues take priority in the acute moment of loss and other needs arise in the course of time. Central needs such as being understood by others [39], social and professional support [40] or creating memories with the children [41] were also confirmed by the available data in this study.

This study only covers the perspective of affected parents. However, many of the needs they express are inherently linked to interactions with HCPs, who also bring their own experiences and needs to the situation. For example, parents in this study point to communication gaps and a lack of empathy, citing routines and ‘daily business’ as reasons for this. Recent studies show different results: Various factors such as institutional conditions, knowledge and expertise, resilience, and marital status influence the development of compassion fatigue [42]. However, it can also arise from emotionally stressful work, lack of support, tension, and failure to achieve one’s own ideals [43]. This shows that affected parents also influence HCPs with their situation, experiences, and needs. However, the responsibility for preventing burnout and compassion fatigue among HCPs lies with healthcare providers.

Bringing the results of this study together with the medium and long-term consequences of pregnancy loss, possible links can be detected. Affected parents have an increased risk of developing anxiety, depression, or post-traumatic stress disorder (PTSD) after their loss [16, 44]. This is causally linked to overwhelming emotions and unmet needs in the early phase, as was also the case in this study. In addition, role changes and a lack of social awareness can lead to the stigmatization of parents [45]. Data analysis clearly shows that grief processes are not a phenomenon that occurs “afterwards.” They arise at the moment of loss and sometimes even before the child loss. At a certain point, parents receive information about an abnormal change in their child or have a premonition themselves that still needs to be confirmed. At this point, a cascade of thoughts set in motion and the overwhelming mental load becomes greater, even though the child has not yet died. This is where the central onset of potential consequences can be located, and early trauma-preventive intervention is needed [17] to adequately support and guide affected parents through this situation.

Several mandates are derived from these results for HCPs. They act as advocates for the parents and stand up for their rights. They must be professionally competent and, simultaneously, be able to communicate empathetically and sensitively. They should recognize the needs of those affected and always communicate openly and honestly with them. At the same time, HCPs should be aware of their own limitations and involve other people such as relatives as social support in their care.

### Limitations

It is important to acknowledge the limitations inherent to this study. Only parents from German-speaking countries (Germany, Austria, Switzerland) were included; people from other countries were not involved. This may be seen as a limitation, as the phenomenon under study is complex and may vary across cultural or even local contexts. On the other hand, the sample of our study is already heterogeneous. Expanding the sample further to include additional countries and cultural contexts may have made it more challenging to generate broadly applicable insights into the experience of this life situation. The quotes are originally in German and have been translated into English for this article. To ensure the accuracy of these quotes, the translation process was carried out by two researchers and checked in turn by a native speaker. Nevertheless, possible misunderstandings from the interviewees’ perspective cannot be completely ruled out. In addition, we were only able to recruit parents aged 30 and over. Younger fathers and mothers did not take part in this study but could potentially have provided different and valuable insights. We will be looking for these in the next stages of the study.

## Conclusion

This study reveals that parents have differentiated and complex needs before and during the intrauterine or perinatal loss of their child. These can also contradict each other or change over time, which is why a regular and individual assessment is necessary.

HCPs are of central importance in supporting parents during the moment of loss and have a major influence in this phase. The experience and needs of parents during this time are closely linked to their medium and long-term outcomes. HCPs can set the course here.

## Supplementary Information

Supplementary Material 1.

## Data Availability

All data supporting the findings of this study are available within the paper.

## Abbreviations

GEHBa: Joint Ethics Committee of Bavarian Universities
HCP: Healthcare professional
SRQR: Standards for Reporting Qualitative Research

## References

[1] GBD. Global, regional, and national stillbirths at 20 weeks’ gestation or longer in 204 countries and territories, 1990–2021: findings from the Global Burden of Disease Study 2021. Lancet. 2024;404(10466):1955–88. 10.1016/S0140-6736(24)01925-1. 39510107 10.1016/S0140-6736(24)01925-1 PMC11694012

[2] Hug L, You D, Blencowe H, Mishra A, Wang Z, Fix MJ, et al. Global, regional, and national estimates and trends in stillbirths from 2000 to 2019: a systematic assessment. Lancet. 2021;398(10302):772–85. 10.1016/S0140-6736(21)01112-0. 34454675 10.1016/S0140-6736(21)01112-0 PMC8417352

[3] Stanton C, Lawn JE, Rahman H, Wilczynska-Ketende K, Hill K. Stillbirth rates: delivering estimates in 190 countries. Lancet. 2006;367(9521):1487–94. 10.1016/S0140-6736(06)68586-3. 16679161 10.1016/S0140-6736(06)68586-3

[4] Kniffka MS, Schöley J, Lee S, Bertens LCM, Been JV, Gunnarsdóttir J. Stillbirth rate trends across 25 European countries between 2010 and 2021: the contribution of maternal age and multiplicity. Eur J Public Health. 2025;35(2):319–27. 10.1093/eurpub/ckae214. 39836899 10.1093/eurpub/ckae214 PMC11967907

[5] Fretts RC. Etiology and prevention of stillbirth. Am J Obstet Gynecol. 2005;193(6):1923–35. 10.1016/j.ajog.2005.03.074. 16325593 10.1016/j.ajog.2005.03.074

[6] Gold KJ, Dalton VK, Schwenk TL, Hayward RA. What causes pregnancy loss? Preexisting mental illness as an independent risk factor. Gen Hosp Psychiatry. 2007;29(3):207–13. 10.1016/j.genhosppsych.2007.02.002. 17484937 10.1016/j.genhosppsych.2007.02.002

[7] Gregory ECW, Barfield WD. U.S. stillbirth surveillance: The national fetal death file and other data sources. Semin Perinatol. 2024;48(1):151873. 10.1016/j.semperi.2023.151873. 38143212 10.1016/j.semperi.2023.151873 PMC11293044

[8] Man J, Hutchinson JC, Heazell AE, Ashworth M, Levine S, Sebire NJ. Stillbirth and intrauterine fetal death: factors affecting determination of cause of death at autopsy. Ultrasound Obstet Gynecol. 2016;48(5):566–73. 10.1002/uog.16016. 27781317 10.1002/uog.16016

[9] Berry SN, Marko T, Oneal G. Qualitative Interpretive Metasynthesis of Parents’ Experiences of Perinatal Loss. J Obstet Gynecol Neonatal Nurs. 2021;50(1):20–9. 10.1016/j.jogn.2020.10.004. 33212051 10.1016/j.jogn.2020.10.004

[10] Bornemisza AY, Javor R, Erdos MB. Exploring Gender Differences in Adult Siblings’ Recollections on Perinatal Loss. Omega (Westport). 2025;91(1):5–25. 10.1177/00302228221131369. 36914968 10.1177/00302228221131369 PMC11894830

[11] Cacciatore J, Bushfield S. Stillbirth: the mother’s experience and implications for improving care. J Soc Work End Life Palliat Care. 2007;3(3):59–79. 10.1300/J457v03n03_06. 18077296 10.1300/J457v03n03_06

[12] Zhao X, Hu H, Zhou Y, Bai Y. What are the long-term effects of child loss on parental health? Social integration as mediator. Compr Psychiatry. 2020;100:152182. 10.1016/j.comppsych.2020.152182. 32430143 10.1016/j.comppsych.2020.152182

[13] Obst KL, Due C, Oxlad M, Middleton P. Men’s grief following pregnancy loss and neonatal loss: a systematic review and emerging theoretical model. BMC Pregnancy Childbirth. 2020;20(1):11. 10.1186/s12884-019-2677-9. 31918681 10.1186/s12884-019-2677-9 PMC6953275

[14] Fernández-Basanta S, Coronado C, Bondas T, Llorente-García H, Movilla-Fernández M-J. Unravelling the grief of involuntary pregnancy loss: a meta-ethnography of midwives’ and nurses’ emotional experiences. Scand J Caring Sci. 2022;36(3):599–613. 10.1111/scs.13028. 34418136 10.1111/scs.13028

[15] Mørk S, Hvidtjørn D, Möller S, Henriksen TB, O’Connor M, Bonanno GA. Grief trajectories after loss in pregnancy and during the neonatal period. J Psychiatr Res. 2023;168:293–9. 10.1016/j.jpsychires.2023.10.052. 37931510 10.1016/j.jpsychires.2023.10.052

[16] Shen Q, Luo X, Wei M, Chen B. Latent trajectories of anxiety and depression among women during subsequent pregnancy following pregnancy loss. J Adv Nurs. 2025;81(4):1875–83. 10.1111/jan.16378. 39105255 10.1111/jan.16378

[17] Benton M, Wittkowski A, Edge D, Reid HE, Quigley T, Sheikh Z, et al. Best practice recommendations for the integration of trauma-informed approaches in maternal mental health care within the context of perinatal trauma and loss: A systematic review of current guidance. Midwifery. 2024;131:103949. 10.1016/j.midw.2024.103949. 38382415 10.1016/j.midw.2024.103949

[18] Givrad S, Wall KM, Goldman LW, Shin JY, Novak EH, Lowell A, et al. A systematic review on the assessment of pregnancy-specific psychological trauma during pregnancy: a call to action. AJOG Glob Rep. 2025;5(1):100451. 10.1016/j.xagr.2025.100451. 40093874 10.1016/j.xagr.2025.100451 PMC11909425

[19] Sterr F, Siepmann J, Nuber-Fischer D, Rester C, Gensheimer K, Bauernfeind L. Parents’ experience of child loss during pregnancy or birth: protocol for an explorative, sequential and participative mixed-methods study. Res Involv Engagem. 2025;11(1):139. 10.1186/s40900-025-00819-8. 41350907 10.1186/s40900-025-00819-8 PMC12681120

[20] O’Brien BC, Harris IB, Beckman TJ, Reed DA, Cook DA. Standards for reporting qualitative research: a synthesis of recommendations. Acad Med. 2014;89(9):1245–51. 10.1097/ACM.0000000000000388. 24979285 10.1097/ACM.0000000000000388

[21] Davidsen AS. Phenomenological Approaches in Psychology and Health Sciences. Qualitative Res Psychol. 2013;10(3):318–39. 10.1080/14780887.2011.608466. 10.1080/14780887.2011.608466 PMC3627202 23606810

[22] Wilson A. A guide to phenomenological research. Nurs Stand. 2015;29(34):38–43. 10.7748/ns.29.34.38.e8821. 25902251 10.7748/ns.29.34.38.e8821

[23] Amineh RJ, Asl HD. Review of Constructivism and Social Constructivism. J Social Sci Literature Lang. 2015;1(1):9–16.

[24] Cobern WW, Constructivism. J Educ Psychol Consultation. 1993;4(1):105–12. 10.1207/s1532768xjepc0401_8.

[25] Clarke PM, Fiebig DG, Gerdtham U-G. Optimal recall length in survey design. J Health Econ. 2008;27(5):1275–84. 10.1016/j.jhealeco.2008.05.012. 18667254 10.1016/j.jhealeco.2008.05.012

[26] Vetter TR, Mascha EJ, Bias. Confounding, and Interaction: Lions and Tigers, and Bears. Oh My! Anesth Analg. 2017;125(3):1042–8. 10.1213/ANE.0000000000002332. 10.1213/ANE.0000000000002332 28817531

[27] Berger PL, Luckmann T. The social construction of reality: a treatise in the sociology of knowledge. Repr. New York: Anchor Books; 1967. http://www.loc.gov/catdir/description/random0414/89018142.html.

[28] Aldridge J. Working with vulnerable groups in social research: dilemmas by default and design. Qualitative Res. 2014;14(1):112–30. 10.1177/1468794112455041.

[29] von der Warth R, Hempler I. Vulnerable Gruppen – Sind wir nicht alle ein wenig verwundbar? Z Evid Fortbild Qual Gesundhwes. 2024;184:1–2. 38218666 10.1016/j.zefq.2023.11.002

[30] World Medical Association. World medical association declaration of Helsinki: ethical principles for medical research involving. Hum Subj JAMA. 2013;310(20):2191–4. 10.1001/jama.2013.281053. 10.1001/jama.2013.281053 24141714

[31] Moser A, Korstjens I, Series. Practical guidance to qualitative research. Part 5: Co-creative qualitative approaches for emerging themes in primary care research: Experience-based co-design, user-centred design and community-based participatory research. Eur J Gen Pract. 2022;28(1):1–12. 10.1080/13814788.2021.2010700. 35037811 10.1080/13814788.2021.2010700 PMC8765256

[32] Brown J. The world café: Shaping our futures through conversations that matter. San Francisco: BK Berrett-Koehler; 2005.

[33] Krueger RA, Designing, and Conducting Focus Group Interviews. 2002. https://www.eiu.edu/ihec/Krueger-FocusGroupInterviews.pdf. Cited 2024 Jul 30.

[34] Braun V, Clarke V. Using thematic analysis in psychology. Qualitative Res Psychol. 2006;3(2):77–101. 10.1191/1478088706qp063oa.

[35] Braun V, Clarke V. What can thematic analysis offer health and wellbeing researchers? Int J Qual Stud Health Well-being. 2014;9:26152. 10.3402/qhw.v9.26152. 25326092 10.3402/qhw.v9.26152 PMC4201665

[36] Braun V, Clarke V. Can I use TA? Should I use TA? Should I not use TA? Comparing reflexive thematic analysis and other pattern-based qualitative analytic approaches. Couns Psychother Res. 2021;21(1):37–47. 10.1002/capr.12360.

[37] Braun V, Clarke V. Supporting best practice in reflexive thematic analysis reporting in Palliative Medicine: A review of published research and introduction to the Reflexive Thematic Analysis Reporting Guidelines (RTARG). Palliat Med. 2024;38(6):608–16. 10.1177/02692163241234800. 38469804 10.1177/02692163241234800 PMC11157981

[38] Heazell AEP, Siassakos D, Blencowe H, Burden C, Bhutta ZA, Cacciatore J, et al. Stillbirths: economic and psychosocial consequences. Lancet. 2016;387(10018):604–16. 10.1016/S0140-6736(15)00836-3. 26794073 10.1016/S0140-6736(15)00836-3

[39] Boyle FM, Mutch AJ, Barber EA, Carroll C, Dean JH. Supporting parents following pregnancy loss: a cross-sectional study of telephone peer supporters. BMC Pregnancy Childbirth. 2015;15:291. 10.1186/s12884-015-0713-y. 26552446 10.1186/s12884-015-0713-y PMC4640395

[40] Hutti MH. Social and professional support needs of families after perinatal loss. J Obstet Gynecol Neonatal Nurs. 2005;34(5):630–8. 10.1177/0884217505279998. 16227519 10.1177/0884217505279998

[41] Oxlad MJ, Franke EF, Due C, Jaensch LH. Capturing parents’ and health professionals’ experiences of stillbirth bereavement photography: A systematic review and meta-synthesis. Women Birth. 2023;36(5):421–8. 10.1016/j.wombi.2023.03.001. 36878762 10.1016/j.wombi.2023.03.001

[42] Liu S, Han H, Athurupana R, Yang T, Nakatsuka M. Compassion fatigue and compassion satisfaction among nurses/midwives caring parents with pregnancy loss or infertility: a cross-sectional study. Front Public Health. 2025;13:1668647. 10.3389/fpubh.2025.1668647. 41211383 10.3389/fpubh.2025.1668647 PMC12589010

[43] Yang F, Liu D, Fan G. Emotional labor and coping strategies of gynecological nurses in recurrent pregnancy loss care: a qualitative phenomenological study. BMC Nurs. 2025;24(1):234. 10.1186/s12912-025-02884-6. 40025485 10.1186/s12912-025-02884-6 PMC11871658

[44] Westby CL, Erlandsen AR, Nilsen SA, Visted E, Thimm JC. Depression, anxiety, PTSD, and OCD after stillbirth: a systematic review. BMC Pregnancy Childbirth. 2021;21(1):782. 10.1186/s12884-021-04254-x. 34794395 10.1186/s12884-021-04254-x PMC8600867

[45] Freda MC, Devine KS, Semelsberger C. The lived experience of miscarriage after infertility. MCN Am J Matern Child Nurs. 2003;28(1):16–23. 10.1097/00005721-200301000-00005. 12514352 10.1097/00005721-200301000-00005

