# Supplementary material for: Needs of parents during their intrauterine or perinatal child loss – a qualitative multi-method study
Source: BMC Public Health. 2026 May 12;26:1526. doi: 10.1186/s12889-026-27687-5 (PMC13169847; doi:10.1186/s12889-026-27687-5)
Supplement: Supplementary file 1 — Supplementary Material 1. [file 12889_2026_27687_MOESM1_ESM.pdf]

Supplement 1: Interview guide for focus group

Running order (semi-structured, questions can be asked flexibly)

1) **Welcome** and information on the purpose of the interview
2) **Introductory question**: Please think back to the loss of your child, your stillbirth. What needs did you experience in this situation?
3) **Topic 1 - Information on the loss**
    a. How were you informed of the (potential) loss of your child?
    b. What needs and requirements did you have at that time?
    c. Who accompanied you during this phase?
4) **Topic 2 – Birth**
    a. What needs (physical/mental/psychological) did you have before and during the birth of your child?
    b. How did the immediate care after birth take place?
    c. How were you treated after birth?
5) **Topic 3 - Dealing with the situation**
    a. How did you deal with the loss?
    b. What were you doing/thinking at that moment?
    c. What did you do to deal with the situation?
    d. What external support have you received?
6) **Topic 4 – Expectations** - What expectations did you have of:
    a. Nurses
    b. Physicians
    c. Midwives
    d. The respective setting (hospital, birth center)
7) **Topic 5 – Ideal care**
    a. In your opinion, what would ideal care/support look like before and during the birth of your stillborn child?
    b. What requirements must be met?
    c. If you could make one suggestion to the care staff to improve the care/support, what would it be?
8) **Closing questions**
    a. What do you remember most positively about the process?
    b. What situation or gesture was most important and helpful for you?
